# Assessment of Pharmacological Interactions between SIRT2 Inhibitor AGK2 and Paclitaxel in Different Molecular Subtypes of Breast Cancer Cells

**DOI:** 10.3390/cells11071211

**Published:** 2022-04-04

**Authors:** Anna Wawruszak, Jarogniew Luszczki, Arkadiusz Czerwonka, Estera Okon, Andrzej Stepulak

**Affiliations:** 1 Department of Biochemistry and Molecular Biology, Medical University of Lublin, 20-093 Lublin, Poland; arkadiusz.czerwonka@umlub.pl (A.C.); estera.okon@umlub.pl (E.O.); andrzej.stepulak@umlub.pl (A.S.); 2 Department of Pathophysiology, Medical University of Lublin, 20-090 Lublin, Poland; jarogniew.luszczki@umlub.pl

**Keywords:** paclitaxel (PAX), AGK2, SIRT2 inhibitor, sirtuin inhibitor (SIRTi), histone deacetylase inhibitor (HDI), breast cancer, pharmacological interactions, isobolographic analysis

## Abstract

Breast carcinoma (BC) is the most commonly diagnosed type of cancer in women in the world. Although the advances in the treatment of BC patients are significant, numerous side effects, severe toxicity towards normal cells as well as the multidrug resistance (MDR) phenomenon restrict the effectiveness of the therapies used. Therefore, new active compounds which decrease the MDR, extend disease-free survival, thereby ameliorating the effectiveness of the current treatment regimens, are greatly needed. Histone deacetylase inhibitors (HDIs), including sirtuin inhibitors (SIRTi), are the epigenetic antitumor agents which induce a cytotoxic effect in different types of cancer cells, including BC cells. Currently, combined forms of therapy with two or even more chemotherapeutics are promising antineoplastic tools to obtain a better response to therapy and limit adverse effects. Thus, on the one hand, much more effective chemotherapeutics, e.g., sirtuin inhibitors (SIRTi), are in demand; on the other hand, combinations of accepted cytostatics are trialed. Thus, the aim of our research was to examine the combination effects of a renowned cytotoxic drug paclitaxel (PAX) and SIRT2 inhibitor AGK2 on the proliferation and viability of the T47D, MCF7, MDA-MB-231, MDA-MB-468, BT-549 and HCC1937 BC cells. Moreover, cell cycle arrest and apoptosis induction were explored. The type of pharmacological interactions between AGK2 and PAX in different molecular subtypes of BC cells was assessed using the advanced isobolographic method. Our findings demonstrated that the tested active agents singly inhibited viability and proliferation of BC cells as well as induced cell cycle arrest and apoptosis in the cell-dependent context. Additionally, AGK2 increased the antitumor effect of PAX in most BC cell lines. We observed that, depending on the BC cell lines, the combinations of tested drugs showed synergistic, additive or antagonistic pharmacological interaction. In conclusion, our studies demonstrated that the consolidated therapy with the use of AGK2 and PAX can be considered as a potential therapeutic regimen in the personalized cure of BC patients in the future.

## 1. Introduction

Female breast cancer (BC) was the main reason of cancer incidence in 2020, with approximately 2.3 million new cases, which represent almost 20% of all cancer patients worldwide. In addition, BC was the fifth most common reason of death due to cancers globally, with more than 650,000 deaths only in 2020. Among women, BCs give rise to one in four cancer cases and one in six cancer deaths, putting BC in the first place in most countries all over the world [1,2,3]. 

BC is a very diversified disease with alternating morphological features, behavior, and response to anticancer therapy [4,5,6]. There are five main molecular subtypes of BC based on the inherency of the estrogen (ER) [4], progesterone (PR) [7] and human epidermal growth factor (HER2) [8] receptors, as well as the intensity of proliferation index-67 (Ki-67) [9], including luminal A (ER+/PR+; HER2−; Ki67−) [10], luminal B ((ER+/PR+; HER2−; Ki67+)/(ER+/PR+; HER2+; Ki67+)) [11], HER2-overexpressed (ER−/PR−; HER2+) [12], triple-negative breast cancer (TNBC) (ER−/PR−; HER2−) [13] and normal-like BC (ER+/PR+; HER2−; Ki67−) [14,15]. 

Molecular profiling represents the earliest attempt to provide a personalized approach to the BC patients’ therapy [14,15,16]; however, despite intense studies and meaningful advances in the treatment of BC, the pathogenesis of this disease is still faintly known, and efficacious forms of therapy of BC remain a challenge. Moreover, significant toxicity towards normal cells, serious adverse effects as well as the MDR phenomenon [17,18] restrain the successful therapy of BC patients [19]. Thus, novel potential drugs which can decrease the emergence of MDR, ameliorate the effectiveness of the currently used treatment methods and prolong disease-free survival (DFS) are greatly desired [19,20].

An incorrect profile of histone acetylation drives many cellular disturbances [21], including induction of cancer initiation and progression [22,23]. It has been demonstrated that abnormalities of histone acetylation are a significant element in the development of BC [21,24]. Activity balance between opposite enzymes, histone acetylases (HATs) [25] and histone deacetylases (HDACs) [26], seems essential to keep the epigenetic regulation of gene expression. Because of homology to silent information regulator 2 (SIR2) in *Saccharomyces cerevisiae*, class III of HDACs was named sirtuins [27] and encompasses SIRT1–SIRT7 [27]. Histone deacetylase inhibitors (HDIs) [28,29], including sirtuin inhibitors (SIRTi), are able to induce cell death and inhibit the proliferation of cancer cells refractory to various cytostatics through regulation of the expression of several genes [20,30].

**Figure 1:**
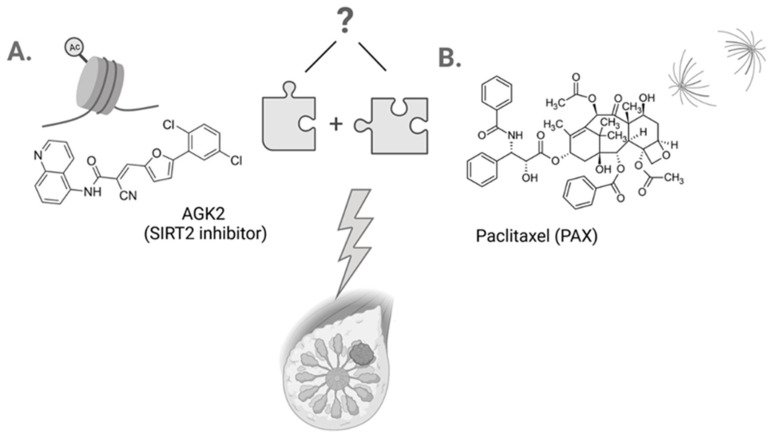
Chemical structures of AGK2 (SIRT2 inhibitor) (C_23_H_13_Cl_2_N_3_O_2_) (**A**) and paclitaxel (PAX) (C_47_H_51_NO_14_) (**B**). Created with www.biorender.com (accessed on 31 March 2022).

AGK2 (C_23_H_13_Cl_2_N_3_O_2_; 2-cyano-3-[5-(2,5-dichlorophenyl)-2-furanyl]-N-5-quinolinyl-2-propenamide) is a potent, cell-permeable, selective SIRT2 inhibitor that minimally affects both SIRT1 and SIRT3 (Figure 1A) [31,32,33]. The mechanism of AGK2 activity based on SIRT2 inhibition leads to the induction of apoptosis, growth arrest and increase in the *PUMA*, *NOXA* and *GADD45* p53-inducible gene expression in glioblastoma cells. Moreover, it has been demonstrated that AGK2 suppresses the formation of spheres in CD133-positive cells isolated from patients’ samples [34,35]. AGK2 also causes cell cycle arrest in the G1 phase, which is mediated via inhibition of the cyclin D1, cyclin-dependent kinase (CDK) 4 and CDK6 expression, as well as inhibited growth and formation of colonies in HeLa cervical cells [36]. Additionally, AGK2 showed an additive effect on the toxicity caused by lapatinib in both the 6–10B sensitive and the 5–8F resistant nasopharyngeal carcinoma (NPC) cells [37] and synergistic interaction with dichloroacetic acid (DCA) in the A549 and H1299 lung cancer cell lines [38]. These findings suggest that AGK2 is able to overcome the drug resistance problem. Given the fact that other HDIs induce a minimal cytotoxic effect regarding normal human cells and are well-tolerated by patients [39], the use of AGK2 individually or combined with other chemotherapeutic drugs may find a potential application in BC therapy.

Paclitaxel (PAX) is one the best known antimitotic chemotherapeutic drugs used in the therapy of different malignancies (Figure 1B) [40]. PAX suppresses the polymerization of microtubules which leads to the inhibition of mitosis and, as a consequence, the activation of mitotic checkpoints [41]. PAX is often applied as the first-line chemotherapeutic in the therapy of BC patients (especially of the TNBC subtype) [42]. Unfortunately, the resistance of BC patients to PAX is a big obstacle in the clinical application of the drug and an important cause of death due to failure of the treatment [43]. A great challenge in the application of PAX in the combined therapy is to limit adverse effects and increase the efficiency of the drug [44].

There is no research on the activity of AGK2 used singly or combined with other compounds in BC models. There is a tremendous knowledge loophole in this research area. Therefore, the present study aimed to investigate cytotoxic, antiproliferative, proapoptotic and cell cycle arrest effects of AGK2 applied individually or combined with PAX in the experimental treatment of various subtypes of BC cells (Figure 1). Additionally, we tested if the combination of AGK2 and PAX will have a stronger effect on BC cells than both compounds applied individually. Moreover, we determined types of pharmacological interaction between AGK2 and PAX using an advanced isobolographic analysis.

## 2. Materials and Methods

### 2.1. SIRT2 Molecular Characteristics

Expression, correlation and mutation data for SIRT2 in BC were extracted from the cBioPortal database (http://www.cbioportal.org/public-portal, accessed on 31 March 2022) and the Human Protein Atlas (https://www.proteinatlas.org/public-portal, accessed on 31 March 2022). The structure of SIRT2 was created using the COSMIC database (https://cancer.sanger.ac.uk/cosmic, accessed on 31 March 2022).

### 2.2. Drugs

PAX and AGK2 were obtained from Sigma-Aldrich (St. Louis; MO, USA) and dissolved in dimethyl sulfoxide (DMSO) at 1 mM and 10 mM (warmed) concentration, respectively. Both drugs were diluted in the recommended culture medium before use.

### 2.3. Cell Lines 

The T47D (ATCC©HTB-133™), MCF7 (ATCC©HTB-22™), MDA-MB-231 (ATTC©HTB-26^TM^), MDA-MB-468 (ATTC©HTB-132^TM^), BT-549 (ATTC©HTB-122^TM^) and HCC1937 (ATTC©CRL-2336^TM^) BC cell lines as well as MCF-10A (ATTC©CRL-10317^TM^) normal breast cells were obtained from the American Type Culture Collection (ATTC) (Manassas; VA, USA). Normal human primary fibroblast culture (HSF) was obtained by the outgrowth technique from skin explants of a young person using a method routinely used in our lab (local ethics committee permission No. KE0254/298/2015). BC cells were maintained in the DMEM/HAM F12 culture medium (Sigma-Aldrich). The medium comprised 10% FBS and antibiotics: penicillin—100 IU/mL, streptomycin—100 µg/mL. The cancer cell lines for the research were chosen to evaluate the differences in responses to test substances. The selected cell lines used in our experiments are models of a specified molecular subtype of BC which makes the obtained results much more reproducible and comparable with other studies. The detailed characteristics of BC cell lines used in the study are summed up in Table 1. 

### 2.4. SIRT2 ELISA Assay

The quantitative measurement of the SIRT2 protein in the T47D, MCF7, MDA-MB-231, MDA-MB-468, BT-549, HCC1937 human BC cells was performed using a Human SIRT2 SimpleStep ELISA^®^ Kit; Abcam (Cambridge, UK). SimpleStep ELISA^®^ employs an affinity tag labeled capture antibody and a reporter-conjugated detector antibody which immunocapture the sample analyte in the solution. This entire complex (capture antibody/analyte/detector antibody) is immobilized via immunoaffinity of an anti-tag antibody coating the well. To perform the assay, the samples and the standards were added to the wells, followed by the antibody mix. After 1 h incubation at room temperature, the wells were washed three times with 350 µL 1× wash buffer to remove the unbound material; 100 µL of the TMB development solution was added to each well and incubated for 10 min. The color reaction was then stopped by adding 100 µL of a stop solution, completing the color change from blue to yellow. The signal was generated proportionally to the amount of the bound analyte, and the intensity was measured at 450 nm. 

### 2.5. Cell Viability Assay

The T47D, MCF7, MDA-MB-231, MDA-MB-468, BT-549, HCC1937 BC and HSF and MDA10A normal cells were treated with PAX (0.001–1 uM) and AGK2 (0.001–0.5 mM) individually or in mixtures for 96 h. After that, BC cells were incubated with 3-(4,5-dimethylthiazol-2-yl)-2,5-diphenyltetrazolium bromide (MTT) at a concentration of 5 mg/mL for 3 h. The optical density of the metabolized product was measured using an Infinite M200 Pro microplate reader at 570 nm. The dose–response curves (DRC) were created in order to determine IC_50_ for PAX and AGK2 in GraphPad Prism 5.0. The effects of combined treatment with PAX and AGK2 were determined with an isobolographic protocol. 

### 2.6. Classification of the Pharmacological Interaction between PAX and AGK2 with Isobolographic Analysis

Type I isobolographic analysis for collateral and nonparallel concentration–effect curves (CECs) was used to classify the types of interactions between PAX and AGK2 for various BC cell lines. The isobolographic method very precisely classifies pharmacological interactions of drugs used in combination at the constant drug concentration ratio (mostly 1:1). 

The percentage of the inhibition of BC cell viability after PAX and AGK2 administration and the CEC for each isobolographically tested drug in BC cell lines were fitted by means of log-probit analysis. The first step in isobolography was based on the determination of the median inhibitory concentrations (IC_50s_) for PAX or AGK2 when administered separately. Then, the test of parallelism of the CECs for PAX and AGK2 was used to analyze the experimental data with isobolography [51]. Details concerning the test for parallelism of CECs were described previously [52]. In this test, PAX had its CEC non-parallel to that of AGK2 in the MCF7, T47D and BT-549 BC cell lines and, simultaneously, PAX had its CEC collateral to that of AGK2 in the MDA-MB-231, MDA-MB-468 and HCC1937 BC cell lines. Details regarding the isobolographic methodology were described elsewhere [53]. From the IC_50_ values determined experimentally for PAX and AGK2 when applied singly, it was possible to calculate the median additive inhibitory concentrations of the mixture of PAX with AGK2, i.e., concentrations of the two-drug mixture which theoretically inhibit 50% of the viability of cells (IC_50 add_), as presented by Tallarida [54,55]. In the case of nonparallel CECs, the equation for the lower line of additivity at the 50% inhibitory effect for the combination of PAX with AGK2 was as follows: y = IC_50_AGK2_ – (IC_50_AGK2_/(IC_50_PAX_/x)^a/b^), where y is the concentration of AGK2, x is the concentration of PAX and a and b are curve-fitting parameters for AGK2 and PAX, respectively. Likewise, the equation for the upper line of additivity at the 50% inhibitory effect for the combination of PAX with AGK2 was as follows: y = IC_50_AGK2_ ((IC_50_PAX_ − x)/IC_50_PAX_)^a/b^. To compute the a and b curve-fitting parameters, probits of response for AGK2 and PAX administered individually were transformed into the % effect. For nonparallel CECs, the additive interaction is the area bounded by lower and upper isoboles of additivity (for more detail see Tallarida) [54,55]. The experimentally-derived (ex-der) IC_50_ values statistically differ if their points are located out of the region bounded by the lower and upper isoboles. Synergy (supra-additivity) is observed if the ex-der IC_50 mix_ points are placed below the area bounded by the lower and upper isoboles of additivity, whereas antagonism (sub-additivity) is illustrated if the ex-der points are placed above this region. The ex-der IC_50_ values statistically differ if their points are placed significantly out of the line of additivity. Synergy (supra-additivity) is observed if the ex-der IC_50 mix_ points are placed below the isobole of additivity, whereas antagonism (sub-additivity) is illustrated if the ex-der points are placed above this line [54,55]. 

Proportions of PAX and AGK2 in the combination were calculated for the constant concentration ratio of 1:1. The mixtures of PAX with AGK2 concentrations were tested on BC cell lines. The determination of the ex-der IC_50 mix_ at the constant 1:1 ratio was based on the concentration of the two-drug mixture that inhibited 50% of cell viability. Further details describing these concepts were published earlier [51,54,55]. 

### 2.7. Cell Proliferation Assay (ELISA BrdU)

The T47D, MCF7, MDA-MB-231, MDA-MB-468, BT-549 and HCC1937 BC cells were incubated with PAX and AGK2 (1/2 IC_50_ and IC_50_) individually or in combination for 48 h. Synthesis of DNA was determined by measurement of incorporation of 5-bromo-2′-deoxyuridine (BrdU) with the use of the BrdU assay (Roche). For more details see [23]. 

### 2.8. Assessment of Apoptosis Induction

The T47D, MCF7, MDA-MB-231, MDA-MB-468 and BT-549 BC cells were treated with PAX and AGK2 individually or in combination (PAX + AGK2) for 48 h. Then, the cells were collected, washed with PBS, fixed and permeabilized with a Cytofix/Cytoperm solution following the manufacturer’s protocol for the PE Active Caspase-3 Apoptosis Kit (BD). Next, BC cells were rinsed using a perm/wash buffer before staining with PE-conjugated anti-active caspase-3 monoclonal rabbit antibodies. Finally, the cells were analyzed using FACSCalibur (BD) with CellQuest. For more details see [23]. 

### 2.9. Assessment of Cell Cycle Arrest

The MCF7 and MDA-MB-468 BC cells were incubated with PAX and AGK2 individually or in combination (PAX + AGK2) for 48 h. After that, the cells were fixed with 80% ethanol at −20 °C for 24 h. Then, the cells were stained with propidium iodide (PI) with the use of the PI/RNase Staining Buffer (BD) following the producer’s protocol. The acquisition parameters (low mode, 60 events/s, 10,000 events) were recorded. The analysis was performed using Cylchred Version 1.0.2 and WinMDI 2.9 for Windows. For more details see [23]. 

### 2.10. Statistical Analysis

The experimentally derived IC_50_ and IC_50 mix_ values for PAX and AGK2 when administered alone or in a mixture at the 1:1 ratio were determined using log-probit analysis. The unpaired Student’s *t*-test with Welch’s correction was used to statistically compare the exp-der IC_50 mix_ values for the mixture of PAX with AGK2 with their respective theoretical additive IC_50 add_ values as described elsewhere [56]. The obtained results were analyzed using GraphPad Prism 5.0 with one-way ANOVA, Tukey’s post-hoc testing. All the data were depicted as the means ± standard deviation (±SD). Results were statistically relevant if *p* < 0.05 (* *p* < 0.05, ** *p* < 0.01, *** *p* < 0.001). 

## 3. Results

### 3.1. SIRT2 Expression, Survival and Genomic Alterations in Breast Cancer (BC) and Breast Cancer (BC) Cells 

**Figure 2:**
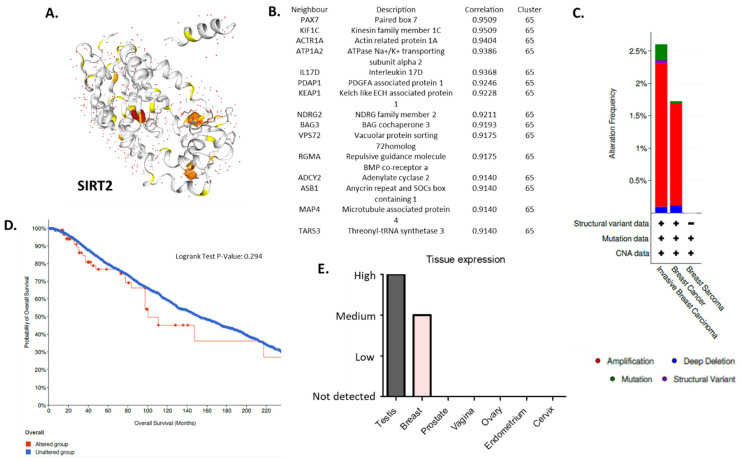
(**A**) Protein structure of SIRT2—NAD-dependent protein deacetylase which deacetylates internal lysines on histone and alpha-tubulin as well as other proteins such as key transcription factors (database: https://cancer.sanger.ac.uk/cosmic3d/protein/SIRT2?pdb=3ZGO, COSMIC database, accessed on 31 March 2022). (**B**) Fifteen nearest protein neighbors of SIRT2 based on tissue RNA expression; the correlation was calculated as Spearman’s correlation. The RNA data were used to cluster genes according to their expression across samples. The resulting clusters were manually annotated to describe the common features in terms of function and specificity. The annotation of the cluster is displayed together with the coincidence score of the gene’s assignment to the cluster. The coincidence is calculated as the fraction of the number of times the gene was assigned to this cluster in repeated calculations and is reported between 0 to 1, where 1 is the highest possible coincidence (Human Protein Atlas, accessed on 31 March 2022). (**C**) Differential alterations (amplification, mutations, deep deletions, structural variants) frequency observed in the *SIRT2* gene in invasive breast carcinoma, breast cancer and breast sarcoma patients—data extracted from TCGA database (cBio database, accessed on 31 March 2022). (**D**) Kaplan–Meier plot: overall patient survival status. The plot indicates no different overall survival of BC patients that harbor at least one alteration in the *SIRT2* gene (red) compared to patients without alterations (blue); 95% confidence interval is shown (cBio database, accessed on 31 March 2022). (**E**) IHC tissue expression of SIRT2 in the reproductive tract system (testes, breast, prostate, vagina, ovaries, endometrium, cervix) (Human Protein Atlas, accessed on 31 March 2022) (**E**). More details: https://cancer.sanger.ac.uk/cosmic, https://www.proteinatlas.org, http://www.cbioportal.org (accessed on 31 March 2022).

**Figure 3:**
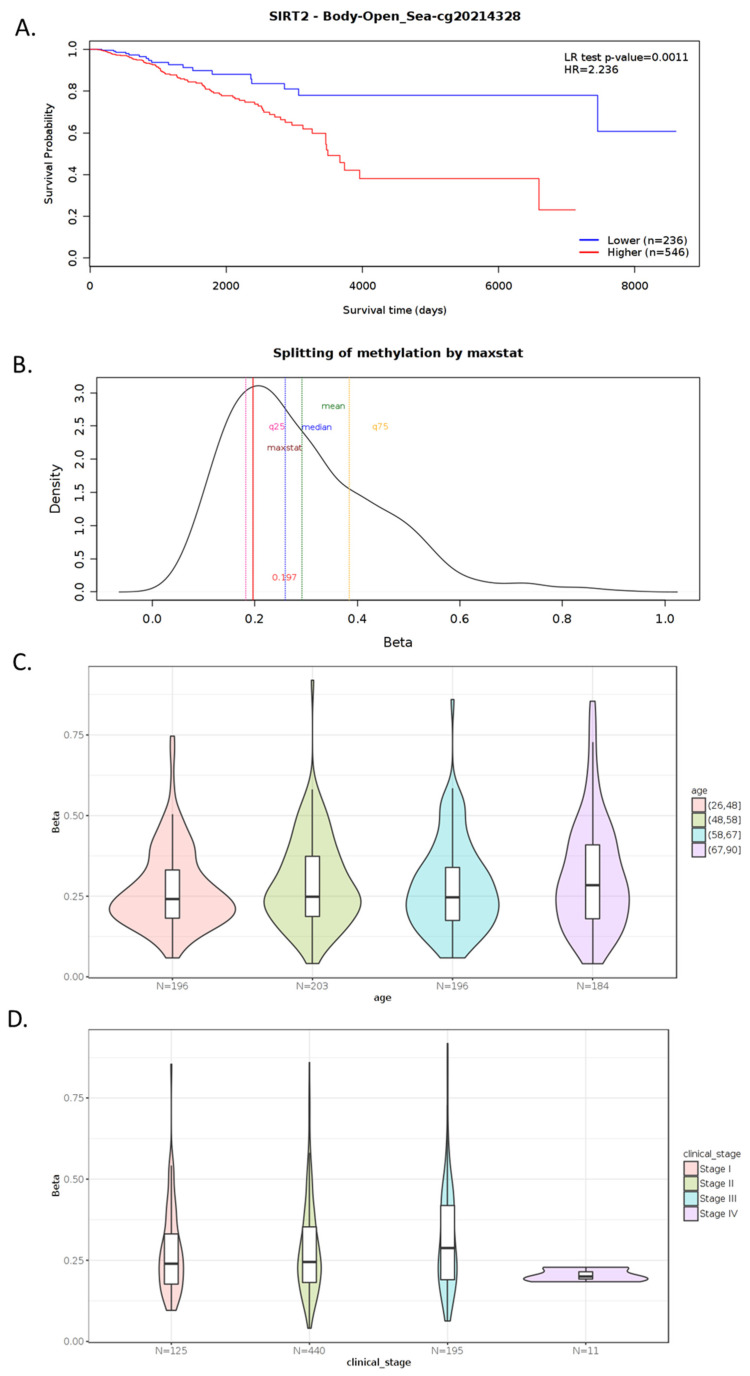
Example of MethSurv graphical outputs generated for CpG cg20214328- SIRT2 in the breast invasive carcinoma samples using a single CpG analysis module. Kaplan–Meier plot showing survival in the higher (shown in red) and lower (shown in blue) methylation groups dichotomized using the maxstat method. The X-axis denotes survival time in days and the Y-axis denotes the probability of patient survival (**A**). Density plot highlighting all the cutoff points evaluated in MethSurv. Different cutoff points are represented by colored texts and the number in red denotes the currently used cutoff point to group the patients (**B**). Violin plots showing the methylation levels among different age groups. Continuous age data are binned into quantiles for visualization (**C**). Violin plots showing the methylation levels among the stage I, II, III and IV BC samples (**D**). A boxplot within each violin plot summarizes the interquartile range and the median methylation levels (shown by a thick black line). The X-axis denotes the patient category while the Y-axis denotes the methylation β-values (ranging from 0 to 1). HR: hazard ratio; LR: log-likelihood ratio; BRCA: breast invasive carcinoma; q25: upper quantile; q75: lower quantile (MethSurv, accessed on 31 March 2022) [57]. More details: https://biit.cs.ut.ee/methsurv/ (accessed on 31 March 2022).

Tissue expression of SIRT2 (Figure 2A) in BC was higher than the expression of SIRT2 in other organs in the reproductive tract system, e.g., prostate, ovaries, vagina, endometrium, cervix (Human Protein Atlas) (Figure 2B,E). The analysis of public genomic databases suggests that approximately 2.6% of invasive breast carcinoma and 1.73% of noninvasive BC samples show patterns of SIRT2 genetic alterations. In both cases, the majority of the alterations are amplifications of SIRT2 (2.21% and 1.57%, respectively) (Figure 2C). However, the Kaplan–Meier plot indicates no significantly different overall survival of BC patients in the altered and unaltered SIRT2 groups despite the fact that, according to the cBio database, the median survival overall was 100.70 months for the altered group and 152.07 months for the unaltered group (95% CI) (Figure 2D). Interestingly, SIRT2 DNA methylation level was associated with overall BC survival (*p*-value = 0.0011) (Figure 3A). Therefore, single CpG methylation patterns of SIRT2 can be a potential biomarker for cancer risk assessment (Figure 3A–D). 

**Figure 4:**
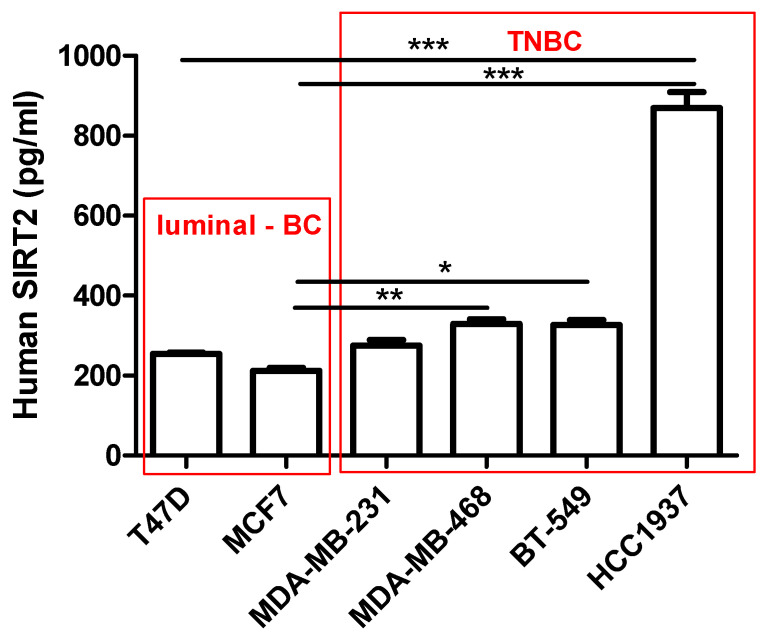
Interpolated concentrations of native SIRT2 (pg/mL) in 100 µg/mL of the protein extracted from the T47D, MCF7, MDA-MB-231, MDA-MB-468, BT-549 and HCC1937 BC cells. The concentrations of SIRT2 were measured in triplicate and interpolated from the SIRT2 standard curve and corrected for sample dilution. The interpolated dilution factor-corrected values are plotted (mean ± SD, *n* = 3; one-way ANOVA, Tukey’s post-hoc testing; * *p* < 0.05, ** *p* < 0.01, *** *p* < 0.001).

SIRT2 concentrations (conc.) (pg/mL) in 100 µg/mL of the protein extracted from the T47D, MCF7, MDA-MB-231, MDA-MB-468, BT-549 and HCC1937 BC cells were determined using the Human SIRT2 Elisa Assay (Figure 4). The conc. of SIRT2 was higher in the TNBC cells (MDA-MB-231 = 275.1 ± 14.3 pg/mL, MDA-MB-468 = 329.4 ± 11.72 pg/mL, BT-549 = 326.9 ± 13.01 pg/mL, HCC1937 = 869.2 ± 40.32 pg/mL, respectively) than in luminal-type BC cells (T47D = 254.5 ± 3.593 pg/mL, MCF7 = 212.0 ± 7.31 pg/mL). The highest conc. of SIRT2 (869.2 ± 40.32 pg/mL) was determined in the most aggressive *BRCA1* and *p53*-mutated HCC1937 TNBC line. 

### 3.2. AGK2 and PAX Administered Individually Decrease the Viability of the T47D, MCF7, MDA-MB-231, MDA-MB-468, BT-549 and HCC1937 BC Cells

**Figure 5:**
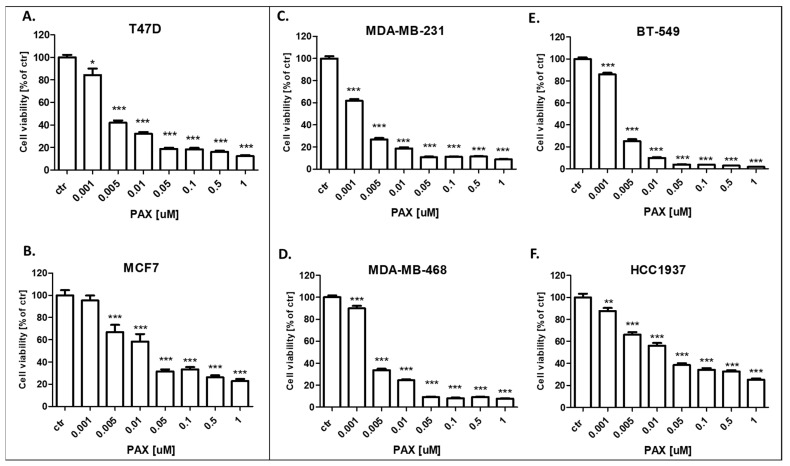
Effect of paclitaxel (PAX) on the viability of the (**A**) T47D, (**B**) MCF7, (**C**) MDA-MB-231, (**D**) MDA-MB-468, (**E**) BT-549 and (**F**) HCC1937 BC cells after 96 h with 0.001–1 μM of the drug in the MTT assay. The data are presented as the means ± standard deviation (±SD); one-way ANOVA, Tukey’s post-hoc testing; * *p* < 0.05, ** *p* < 0.01, *** *p* < 0.001.

**Figure 6:**
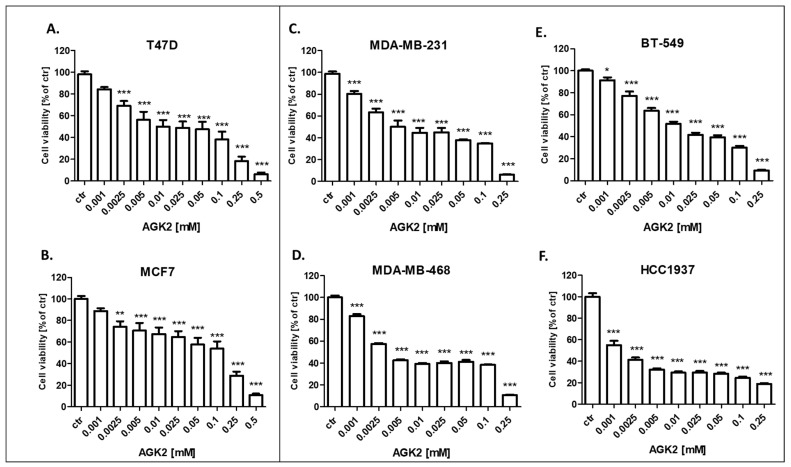
Effect of AGK2 on the viability of the (**A**) T47D, (**B**) MCF7, (**C**) MDA-MB-231, (**D**) MDA-MB-468, (**E**) BT-549 and (**F**) HCC1937 BC cells after 96 h with 0.001–0.5 mM of the drug in the MTT assay. The data are presented as the means ± standard deviation (±SD); one-way ANOVA, Tukey’s post-hoc testing; * *p* < 0.05, ** *p* < 0.01, *** *p* < 0.001.

The cytotoxic activity of PAX and AGK2 for the T47D, MCF7 luminal as well as MDA-MB-231, MDA-MB-468, BT-549 and HCC1937 TNBC cell lines was determined with the use of the MTT assay to calculate the IC_50_ value for both drugs in all the BC cell lines. IC_50_ values ± SEM for each BC cell line were determined according to the log-probit analysis of the concentration–response relationship (CRR) effects of two active agents. The IC_50_ values for the studied BC cell lines are presented in Table 2. All the BC cell lines were exposed to control or increasing concentrations of PAX (0.001–1 µM) and AGK2 (0.001–0.5 mM). PAX and AGK2 administered individually inhibited cell viability in all the BC cell lines in a dose-dependent fashion (Figure 5 and Figure 6). Interestingly, the cytotoxic effect of AGK2 was less evident in the luminal (MCF7, T47D) BC cells than in the MDA-MB-231, MDA-MB-468, BT-549 and HCC1937 TNBC cells. Furthermore, the most sensitive BC cell line for the AGK2 treatment was the most aggressive HCC1937 BRCA1 and p53-mutated TNBC cell line with IC_50_ = 1.326 µM. The IC_50_ value for the most resistant MCF7 luminal BC cell line (66.198 µM) was more than 50 times higher than IC_50_ for the HCC1937 cells (Figure 5). The IC_50_ values for PAX in BC cell lines ranged between 1 and 20 nM. A higher IC_50_ value was calculated for the luminal BC cells (6–16 nM) than for the TNBC cells (1–5 nM), except for the mutant HCC1937 BC cell line (IC_50_ = 18.604 nM) (Figure 5). Interestingly, HCC1937 BC cells were the most sensitive to the AGK2 treatment and the least sensitive to PAX.

### 3.3. AGK2 Slightly Decreases the Viability of Human Skin Fibroblasts (HSF) and MCF-10A Normal Breast Cells

**Figure 7:**
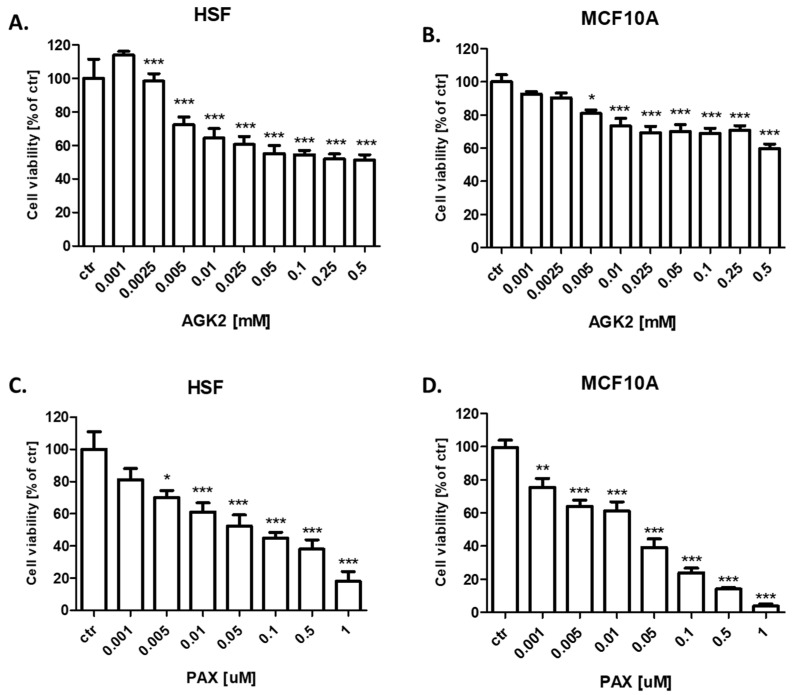
Effect of AGK2 on the viability of (**A**) human skin fibroblasts (HSF) and (**B**) the MCF-10A normal breast cells after 96 h with 0.001–0.5 mM of AGK2 and the effect of PAX on the viability of (**C**) human skin fibroblasts (HSF) and (**D**) the MCF-10A normal breast cells after 96 h with 0.001–1 μM of the drug in the MTT assay. The data are presented as the means ± standard deviation (±SD); one-way ANOVA, Tukey’s post-hoc testing; * *p* < 0.05, ** *p* < 0.01, *** *p* < 0.001.

In our study, we demonstrated that AGK2 decreases the viability of both human skin fibroblasts (HSF) (Figure 7A) and the MCF-10A normal BC cells (Figure 7B) much weaker than in all the analyzed BC cell lines. The decrease in viability did not reach 50% in HSF and the MCF10A normal cells; therefore, it was not possible to determine the IC_50_ values. AGK2 also had a much weaker cytotoxic effect on normal cells than the standard cytotoxic drug PAX in both analyzed normal cell lines (Figure 7C,D).

### 3.4. AGK2 Administered in Combination with PAX Decreases the Viability of the T47D, MCF7, MDA-MB-231, MDA-MB-468, BT-549 and HCC1937 BC Cells

**Figure 8:**
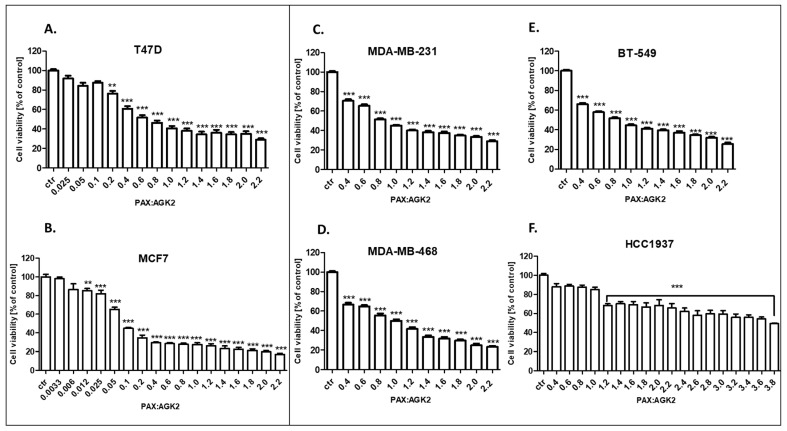
The antiproliferative effect of AGK2 and paclitaxel (PAX) on the (**A**) T47D, (**B**) MCF7, (**C**) MDA-MB-231, (**D**) MDA-MB-468, (**E**) BT-549 and (**F**) HCC1937 BC cells after 96 h with 1:1 drug PAX/AGK2 in the MTT assay. BC cells were treated with PAX and AGK2 using different ratios of IC_50_ (2.0 = IC_50_ + IC_50_). The data are presented as the means ± standard deviation (±SD); one-way ANOVA, Tukey’s post-hoc testing; ** *p* < 0.01, *** *p* < 0.001.

The cytotoxic effect of PAX and AGK2 used together against the T47D, MCF7, MDA-MB-231, MDA-MB-468, BT-549 and HCC1937 BC cells was determined in the MTT assay. PAX and AGK2 were used together with a 1:1 drug mixture in increasing doses. BC cells were treated with the PAX and AGK2 mixture in different ratios of IC_50_ (2.0 means IC_50_ of PAX + IC_50_ of AGK2). Here, we showed the concentration-dependent inhibition of both drugs in a 1:1 combination in all the analyzed BC cell lines (Figure 8). Interestingly, the MCF7 cell line was the most resistant cell line both to PAX (Figure 5) and AGK2 (Figure 6) alone. However, these cells were the most sensitive to the PAX/AGK2 treatment (Figure 8). Similarly, the HCC1937 cells were the most sensitive to the AGK2 treatment alone (Figure 6), but in case of the combination of PAX and AGK2, these cells were the most resistant among all the used BC cell lines (Figure 8). 

### 3.5. AGK2 and PAX Administered Singly and in Combination Decrease Proliferation of the T47D, MCF7, MDA-MB-231, MDA-MB-468, BT-549 and HCC1937 BC Cells

**Figure 9:**
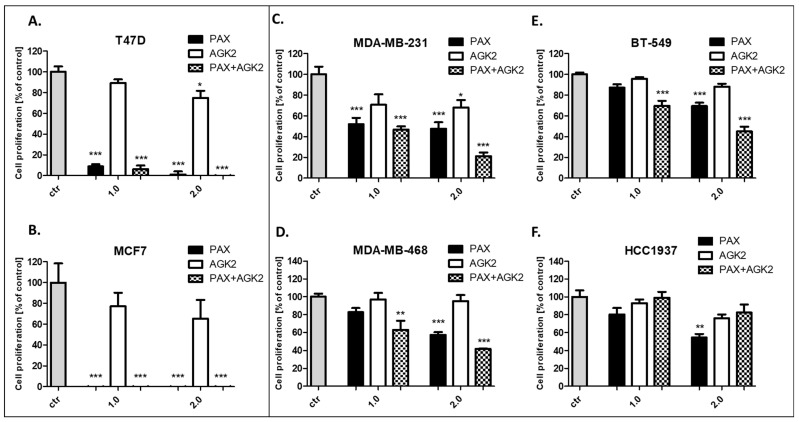
Effect of AGK2 and paclitaxel (PAX) alone or AGK2 in combination with PAX on the (**A**) T47D, (**B**) MCF7, (**C**) MDA-MB-231, (**D**) MDA-MB-468, (**E**) BT-549 and (**F**) HCC1937 BC cells in the BrdU assay. BC cells were incubated for 48 h individually (control) or with the drugs (1.0 = ½ IC_50_; 2.0 = IC_50_ determined in the MTT assay). The data are presented as the means ± standard deviation (±SD); one-way ANOVA, Tukey’s post-hoc testing; * *p* < 0.05, ** *p* < 0.01, *** *p* < 0.001.

The influence of PAX and AGK2 on the proliferation of BC cells was determined in the ELISA BrdU assay. The BrdU test is a non-isotopic immunoassay used for the quantification of BrdU incorporation into freshly synthesized DNA in proliferating cells. The T47D, MCF7, MDA-MB-231, MDA-MB-468, BT-549 and HCC1937 BC cells were exposed to the culture medium (control), PAX and AGK2 individually or PAX with AGK2 in a 1:1 combination (1.0 = ½ IC_50_; 2.0 = IC_50_ determined in the MTT assay). In our studies, PAX reduced the proliferation of all the studied BC cells in a concentration-dependent fashion after 48 h of treatment with PAX. The strongest antiproliferative effect of PAX was observed in the luminal-type BC cells (T47D and MCF7), whereas the BT-549 cell line was the most resistant to the PAX treatment. AGK2 also decreased the proliferation of all BC cells in a concentration-dependent fashion; however, the antiproliferative effect of AGK2 was much weaker than the effect caused by PAX. The T47D and MCF7 cells were the most sensitive for the combined PAX and AGK2 treatment. This effect seems to be due to the very strong activity of PAX in these cell lines. The combination of PAX with AGK2 reduced BC cell proliferation in all the TNBC cell lines (MDA-MB-231, MDA-MB-468, BT-549), except for the HCC1937 cells (Figure 9).

### 3.6. Isobolographic Analysis of the Drug–Drug Interactions between AGK2 and PAX for Nonparallel Concentration–Effect Curves in the T47D, MCF7 and BT-549 BC Cells

**Figure 10:**
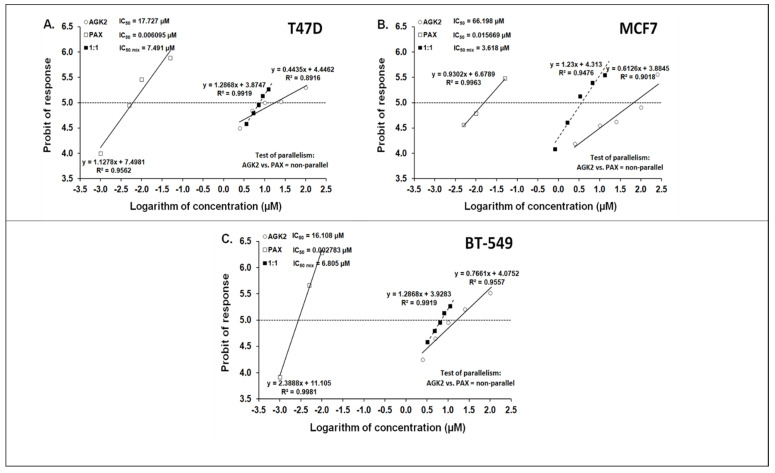
Log-probit concentration–effect relationship curves (CECs) for PAX and AGK2 administered alone and in combination (at the fixed-ratio of 1:1) for the T47D (**A**), MCF7 (**B**) and BT-549 (**C**) cells. Concentrations of PAX and AGK2 when administered individually and the mixture of these drugs in a 1:1 ratio combination were transformed into logarithms, whereas the antiproliferative effects in BC cell lines were measured using the MTT assay and transformed into probits [58]. Linear regression equations of CECs are presented on the graph, where y is the probit of the response, x is the logarithm (to the base 10) of a drug concentration, R2—coefficient of determination. The dotted line parallel to the X-axis and reflecting the fifth probit indicates the approximate IC_50_ values for the studied drugs given alone and the mixture of PAX and AGK2 in a constant ratio of 1:1. Test of parallelism of CECs for PAX and AGK2 indicated that both lines are not parallel to each other.

**Figure 11:**
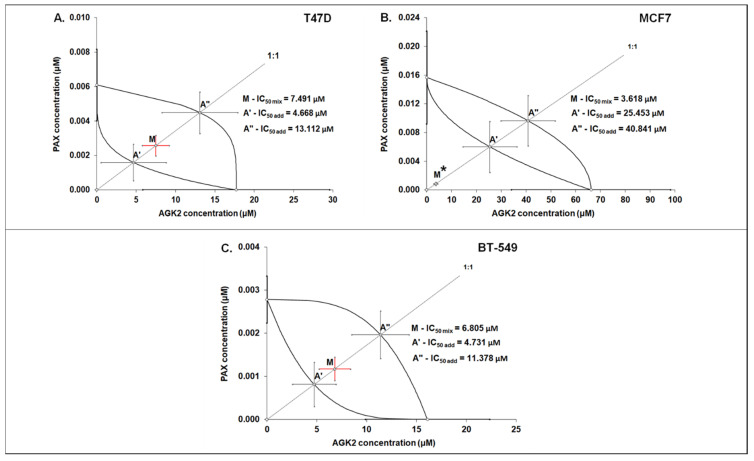
Isobolographic analysis of PAX and AGK2 for nonparallel CECs. Isobolograms illustrate additive and synergistic interactions between PAX and AGK2 in the T47D (**A**), MCF7 (**B**) and BT-549 (**C**) cells measured using the MTT assay. The IC_50_ values for PAX and AGK2 are plotted on the X- and Y-axes. The solid lines on the X- and Y-axes represent the SEM for the IC_50_ values for the studied drugs administered alone. The lower and upper isoboles of additivity represent the curves connecting the IC_50_ values for PAX and AGK2 administered alone. The line starting from the beginning of the Cartesian plot system corresponds to the constant ratio combination of 1:1. The points A’ and A” depict the theoretically calculated IC_50 add_ values for both the lower and upper isoboles of additivity. Point M represents the IC_50 mix_ value for the total concentration of the mixture expressed as proportions of PAX and AGK2 that produced a 50% antiproliferative effect (50% isobole) in the T47D, MCF7 and BT-549 cells measured using the MTT assay. On the graph, the SEM values are presented as horizontal and vertical error bars for every IC_50_ value. The IC_50 mix_ value for BC cell line MCF7 is placed significantly below point A’, indicating a supra-additive (synergistic, Student’s *t*-test with Welch’s correction, * *p* < 0.05) interaction between PAX and AGK2. In contrast, the IC_50 mix_ values for the T47D and BT-549 BC cells are placed close to point A’, indicating an additive interaction between PAX and AGK2 in the T47D and BT-549 cells.

The isobolographic analysis demonstrated that the combination of PAX with AGK2 at the constant ratio of 1:1 produced an additive interaction in the T47D BC cells (Figure 5A and Figure 6A). The IC_50 mix_ value for this combination was 7.491 μM, whereas the additively calculated IC_50 add_ values were 4.668 μM (for the lower IC_50 add_) and 13.112 μM (for the upper IC_50 add_; Table 3), respectively. No significant difference with Student’s *t*-test with Welch’s correction was observed between the IC_50 mix_ and IC_50 add_ values (t = 0.626; df = 245.6; *p* = 0.532; Table 3, Figure 10A and Figure 11A). In contrast, the mixture of PAX with AGK2 in the constant ratio of 1:1 exerted isobolographically a supra-additive (synergistic; * *p* < 0.05) interaction in the MCF7 BC cells (Figure 10B and Figure 11B). The IC_50 mix_ value for this combination was 3.618 μM, whereas the additively calculated IC_50 add_ values were 25.453 μM (for the lower IC_50 add_) and 40.841 μM (for the upper IC_50 add_; Table 3), respectively. Student’s *t*-test with Welch’s correction revealed that the IC_50 mix_ value significantly differed from the IC_50 add_ values (t = 2.003; df = 189.4; *p* = 0.0466; Table 3, Figure 10B and Figure 11B). In the BT-549 BC cells, the mixture of PAX with AGK2 in the constant ratio of 1:1 exerted isobolographically an additive interaction (Figure 10C and Figure 11C). The IC_50 mix_ value for this combination was 6.805 μM, whereas the additively calculated IC_50 add_ values were 4.731 μM (for the lower IC_50 add_) and 11.378 μM (for the upper IC_50 add_; Table 3), respectively. No statistical significance was observed because the IC_50 mix_ value did not differ from the IC_50 add_ values with Student’s *t*-test with Welch’s correction (t = 0.769; df = 274.3; *p* = 0.442; Table 3, Figure 10C and Figure 11C).

### 3.7. Isobolographic Analysis of Interaction between AGK2 and PAX for Parallel Concentration–Effect Curves in the MDA-MB-231, MDA-MB-468 and HCC1937 BC Cells

**Figure 12:**
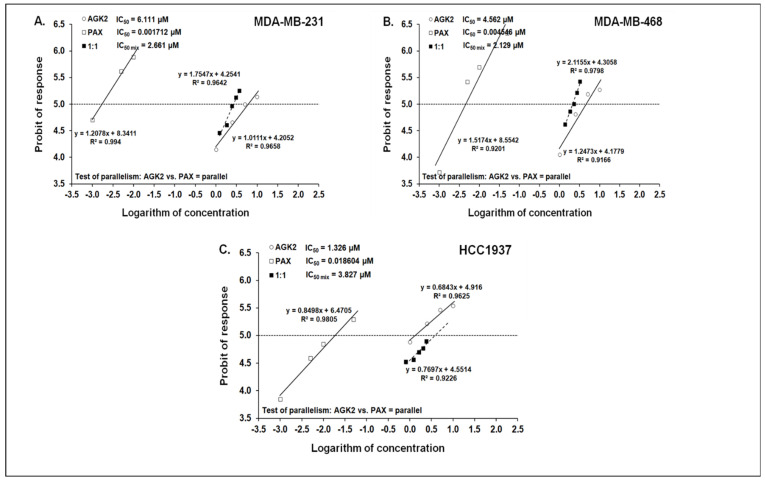
Log-probit concentration–effect relationship curves (CECs) for PAX and AGK2 in the MDA-MB-231 (**A**), MDA-MB-468 (**B**) and HCC1937 (**C**) cells measured using the MTT assay. Concentrations of PAX and AGK2 were administered separately, and the mixture of the drugs (1:1) was transformed into logarithms, whereas the antiproliferative effects produced by the drugs were transformed into probits [58]. Linear regression equations of CECs are presented on the graph where y is the probit of the response, x is the logarithm (to the base 10) of a drug concentration, R2—coefficient of determination. The dotted line indicates the approximate IC_50_ values for the studied drugs given alone and the mixture of PAX and AGK2 in a constant ratio of 1:1. Test of parallelism of CECs for PAX and AGK2 indicated that both lines are mutually collateral.

**Figure 13:**
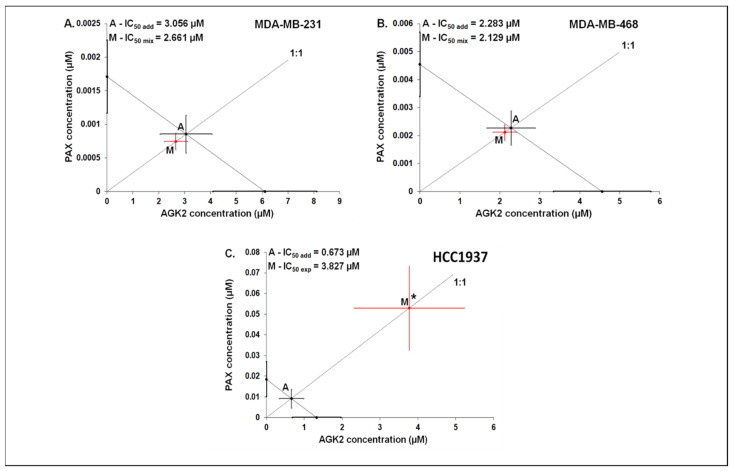
Isobolographic analysis between PAX and AGK2 for collateral CECs. Isobolograms illustrate additive and antagonistic interactions between PAX and AGK2 with respect to their antiproliferative effects in the MDA-MB-231 (**A**), MDA-MB-468 (**B**) and HCC1937 (**C**) cells. The IC_50_ values for PAX and AGK2 are plotted graphically on the X- and Y-axes, respectively. The solid lines on the X- and Y-axes represent the SEM for the IC_50_ values for the studied drugs administered alone. The diagonal line connecting the IC_50_ values for PAX and AGK2 illustrates the isobole of additivity. The line starting from the beginning of the Cartesian plot system corresponds to the constant ratio of 1:1 for the combination of the drugs. Point A depicts the theoretically calculated IC_50 add_ value for the isobole of additivity. Point M represents the IC_50 mix_ value for the total concentration of the mixture expressed as proportions of PAX and AGK2 that produced a 50% antiproliferative effect (50% isobole). On the graph, the SEM values are presented as horizontal and vertical error bars for every IC_50_ value. The IC_50 mix_ value for the BC cell line HCC1937 is placed significantly above point A, indicating a sub-additive (antagonistic) interaction (Student’s *t*-test with Welch’s correction; * *p* < 0.05) between PAX and AGK2. In contrast, the IC_50 mix_ values for the BC cell lines MDA-MB-231 and MDA-MB-468 are placed close to point A, indicating an additive interaction between PAX and AGK2 in the MDA-MB-231 and MDA-MB-468 BC cells.

The mixture of PAX with AGK2 in the constant ratio combination of 1:1 demonstrated an additive interaction in the MDA-MB-231 BC cells (Figure 12A and Figure 13A). The IC_50 mix_ value for this combination was 2.661 μM, whereas the additively calculated IC_50 add_ value was 3.056 μM (Table 4). No significance was reported between the IC_50 mix_ and IC_50 add_ values with Student’s *t*-test with Welch’s correction (t = 0.359; df = 222.9; *p* = 0.720; Table 4, Figure 12A and Figure 13A). Similarly, the mixture of PAX with AGK2 in the constant ratio of 1:1 produced an additive interaction in the MDA-MB-468 BC cells (Figure 12B and Figure 13B). The IC_50 mix_ value for this combination was 2.129 μM, whereas the additively calculated IC_50 add_ value was 2.283 μM (Table 4). With Student’s *t*-test with Welch’s correction, no significance was reported since the IC_50 mix_ value did not differ from the IC_50 add_ value (t = 0.227; df = 232.7; *p* = 0.824; Table 4, Figure 12B and Figure 13B). In contrast, the mixture of PAX with AGK2 in the constant ratio of 1:1 exerted a sub-additive (antagonistic; * *p* < 0.05) interaction in the HCC1937 BC cells ( 12C and 13C). The IC_50 mix_ value for this two-drug combination was 3.827 μM, whereas the additively calculated IC_50 add_ value was 0.673 μM (Table 4). The IC_50 mix_ value significantly differed from the IC_50 add_ value with Student’s *t*-test with Welch’s correction (t = 2.087; df = 130.7; *p* = 0.0389; Table 4, Figure 12C and Figure 13C).

### 3.8. AGK2 and PAX Administered Singly and in Combination Induce Apoptosis of the T47D, MCF7, MDA-MB-231, MDA-MB-468 and BT-549 BC Cells

**Figure 14:**
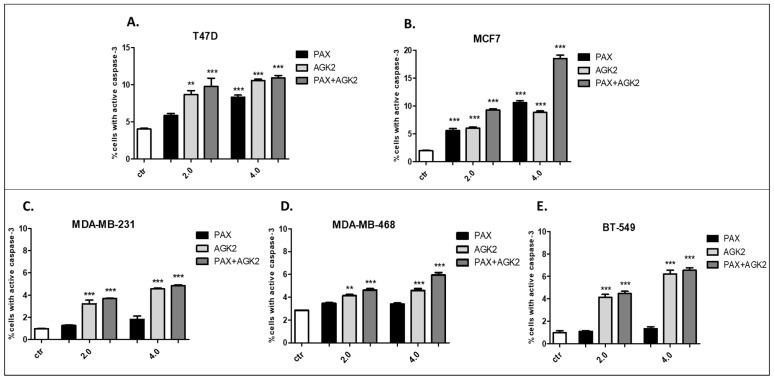
Effect of AGK2 and paclitaxel (PAX) alone or AGK2 in combination with PAX on caspase-3 activation in the (**A**) T47D, (**B**) MCF7, (**C**) MDA-MB-231, (**D**) MDA-MB-468 and (**E**) BT-549 BC cells. BC cells were exposed to the PAX/AGK2 treatment for 48 h using selected ratios of the IC_50_ determined in the MTT assay (2.0 = IC_50_ + IC_50_, 4.0 = 2IC_50_ + 2IC_50_) and analyzed by FACS. The data are presented as the means ± standard deviation (±SD); one-way ANOVA, Tukey’s post-hoc testing; ** *p* < 0.01, *** *p* < 0.001.

**Figure 15:**
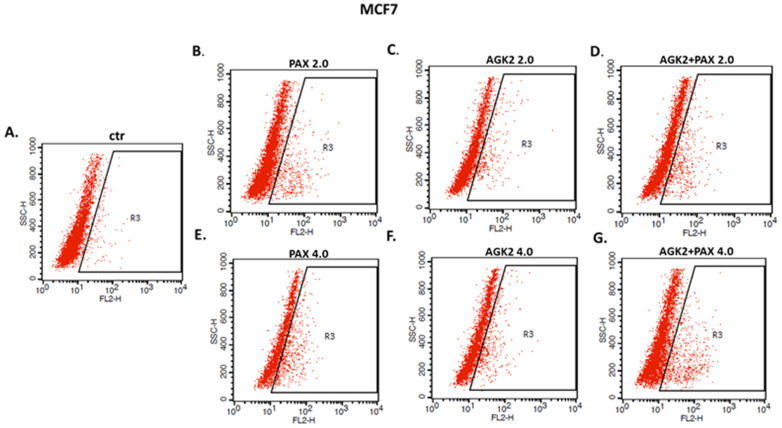
Representative dot plots from the FACS analysis of the MCF7 luminal-type BC cells after a 48 h incubation with a medium (ctr) (**A**), paclitaxel (PAX) (**B**,**E**), AGK2 (**C**,**F**) and PAX + AGK2 (**D**,**G**) (2.0 = IC_50_ + IC_50_, 4.0 = 2IC_50_ + 2IC_50_). R3-apoptotic cells with active caspase-3.

**Figure 16:**
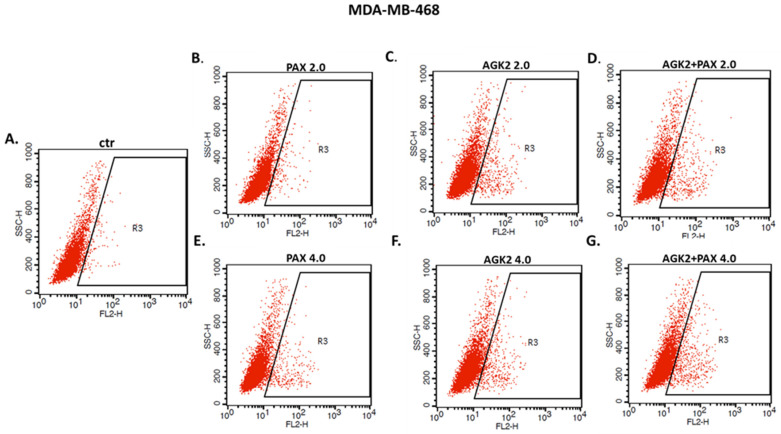
Representative dot plots from the FACS of the MDA-MB-468 TNBC cells after a 48 h incubation with a medium (ctr) (**A**), paclitaxel (PAX) (**B**,**E**), AGK2 (**C**,**F**) and PAX + AGK2 (**D**,**G**) (2.0 = IC_50_ + IC_50_, 4.0 = 2IC_50_ + 2IC_50_). R3—apoptotic cells with active caspase-3.

The influence of PAX and AGK2 applied alone or together on the induction of apoptosis was determined as a number of cells with active caspase-3 and analyzed by FACS (Figure 14). The T47D, MCF7, MDA-MB-231, MDA-MB-468 and BT-549 BC cells were exposed to an individual or concomitant PAX and AGK2 treatment for 48 h using selected ratios of the IC_50_ determined in the MTT assay (2.0 = IC_50_ + IC_50_, 4.0 = 2IC_50_ + 2IC_50_). The PAX and AGK2 used individually increased the number of cells with active caspase-3 versus control in the selected cell lines in a concentration-dependent fashion (Figure 14, Figure 15 and Figure 16). PAX and AGK2 used together slightly increased the percentage of apoptotic cells, suggesting that AGK2 gently strengthens the effect of PAX (Figure 14). The most evident increase in the number of apoptotic cells after the PAX and AGK2 treatment both separately and in combination was observed in the MCF7 luminal BC cells (Figure 15).

### 3.9. AGK2 and PAX Administered Singly and in Combination Induce Cell Cycle Arrest in the MCF7 and MDA-MB-468 BC Cells in a Cell-Dependent Manner

**Figure 17:**
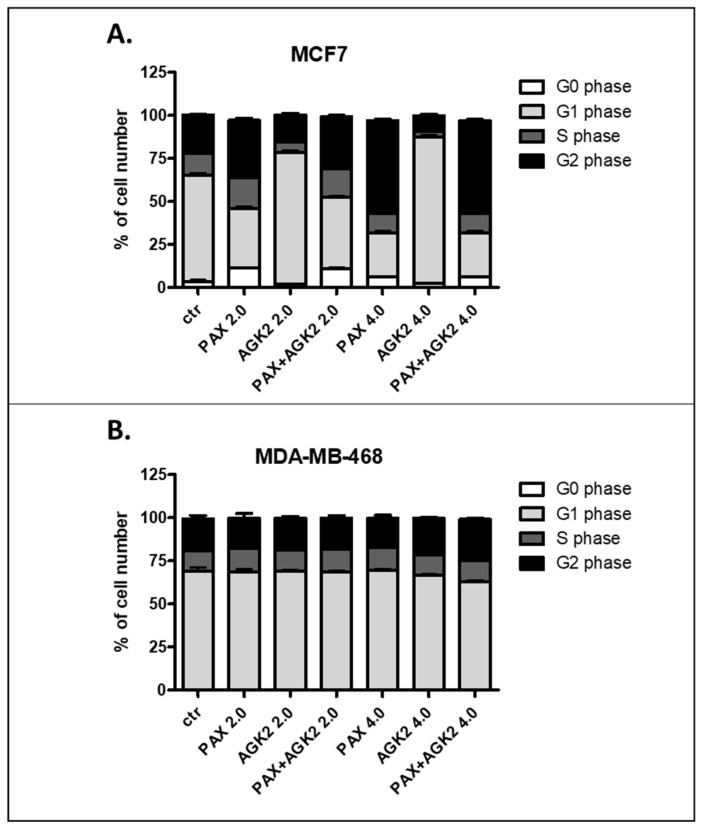
Effect of AGK2 and paclitaxel (PAX) alone or AGK2 combined with PAX on cell cycle progression in the (**A**) MCF7 luminal and (**B**) MDA-MB-468 TNBC cells. BC cells were exposed to individual or concomitant PAX and AGK2 treatment for 48 h using selected ratios of the IC_50_ determined in the MTT assay (2.0 = IC_50_ + IC_50_, 4.0 = 2IC_50_ + 2IC_50_), stained with propidium iodide (PI) and analyzed by means of FACS. The data are presented as the means ± standard deviation (±SD).

**Figure 18:**
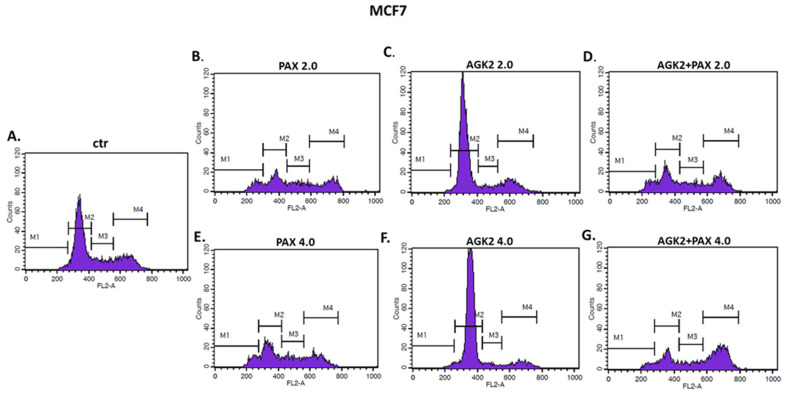
Representative histograms from the flow cytometry analysis of the MCF7 luminal-type breast cancer (BC) cells after a 48 h incubation with a medium (ctr) (**A**), paclitaxel (PAX) (**B**,**E**), AGK2 (**C**,**F**) and PAX + AGK2 (**D**,**G**) (2.0 = IC_50_ + IC_50_, 4.0 = 2IC_50_ + 2IC_50_). M1—G0 (pre-G1) phase; M2—G1 phase; M3—S phase; M4—G2 phase.

**Figure 19:**
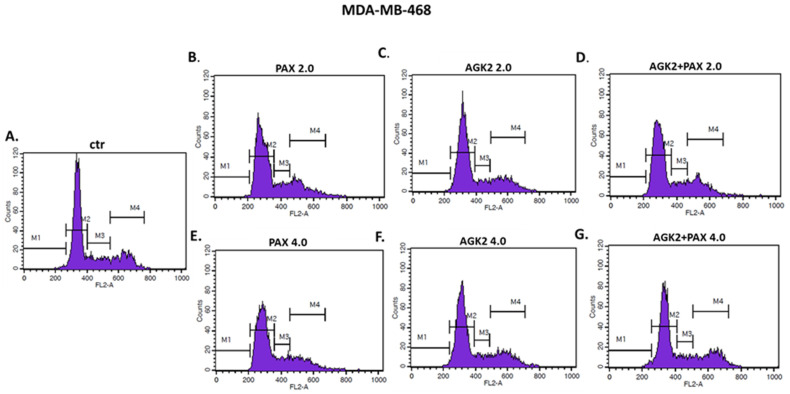
Representative histograms from the flow cytometry analysis of the MDA-MB-468 TNBC cells after a 48 h incubation with a medium (ctr) (**A**), paclitaxel (PAX) (**B**,**E**), AGK2 (**C**,**F**) and PAX + AGK2 (**D**,**G**) (2.0 = IC_50_ + IC_50_, 4.0 = 2IC_50_ + 2IC_50_). M1—G0 (pre-G1) phase; M2—G1 phase; M3—S phase; M4—G2 phase.

Since a decrease in BC cells proliferation resulted from an inhibition of cell division, cell cycle progression analysis by means of FACS was performed. The effect of the PAX and AGK2 treatment (individually or together) on cell cycle arrest was examined in two (luminal-type MCF7 and TNBC MDA-MB-468) BC cell lines. FACS analysis of PI-stained BC cells demonstrated that the treatment of the MCF7 BC cells with PAX separately for 48 h leads to the accumulation of BC cells in the pre-G1 and G2 phases in a concentration-dependent fashion. Interestingly, incubation of the MCF7 cells with AGK2 caused cell cycle inhibition in the G1 phase. Concomitant treatment with PAX and AGK2 demonstrated a tendency similar to the PAX treatment—accumulation of cells in the pre-G1 and G2 phases (Figure 17). This effect was much more evident in the MCF7 luminal BC cells (Figure 17 and Figure 18) than in the TNBC MDA-MB-468 cells (Figure 17 and Figure 19), where the changes in the course of cell cycle were not so manifest. 

## 4. Discussion

BC is a very diverse disease with an enormous genetic and phenotypic variation. All this heterogeneity makes the BC patients’ treatment difficult. Thus, the choice of an appropriate form of BC therapy is highly important [44]. 

PAX is a commonly known cytotoxic agent used in the treatment of many types of cancers, e.g., BC. This antimitotic chemotherapeutic suppresses the polymerization of microtubules, which leads to the activation of mitotic checkpoints and, consequently, induction of apoptosis [44,59]. Despite the fact that PAX is currently widely used in BC treatment, its effectiveness is limited by serious adverse effects (e.g., cardiotoxicity, neurotoxicity, hematological toxicity). Moreover, because of poor solubility, PAX needs to be prepared in a solvent based on lipids and dehydrated ethanol. Unfortunately, this vehicle can cause sensory neuropathy, histamine-mediated hypersensitivity reactions (e.g., hypotension, dyspnea, bronchospasm, chest pain, urticaria, erythematous rash) or impairment of drug delivery [44]. The resistance of BC cells to PAX is another big obstacle in the application of the drug in clinics and an important cause of patients’ death linked with failure of the therapy. The ABCB and P-gp proteins play an important role in the tolerance of BC cells to PAX. Both these molecules take part in the efflux of PAX outside of cancer cells [44,60]. 

The aforementioned limitations of the PAX use, which include serious side effects, limited solubility and chemoresistance, push scientists towards the search for a more effective combined targeting form of BC therapies, in which PAX will play an essential role. It has been demonstrated that posttranslational modifications act as positive regulators and induce the transcriptional activity of the *FOXK2* gene which consequently strengthens the cytotoxic response to PAX [44]. 

A balance in the opposite activity of HATs and HDACs plays a crucial role in the epigenetic control of gene expression. The impairment in the equilibrium between HAT and HDAC activities is associated with the emergence of BC. HDIs maintain the proper acetylation profile in cells through targeting both histone and non-histone proteins, and as consequence reverse the function of proteins that take part in BC progression [61]. Sirtuins (SIRTs) are among the key enzymatic proteins engaged in the development and metastasis of different types of neoplasms, e.g., BC. SIRTs are crucial regulators in a variety of different cellular and physiological processes, e.g., genome stability, cell survival, cell proliferation and differentiation, DNA damage, stress response, aging, energy homeostasis, metabolism, organ development as well as cancer progression [62]. The participation of SIRT2 in the process of tumorigenesis has been vastly studied in BC. It has been noticed that SIRT2 can act as a tumor suppressor, but only in early BC carcinogenesis; inversely, in advanced stages of cancer, overexpression of SIRT2 is associated with a more aggressive phenotype [63]. SIRT2 has been reported to be highly expressed and frequently amplified in basal-like breast cancer (BLBC). The SLUG protein has been found to be a deacetylase target of SIRT2, and SIRT2 overexpression promotes SLUG stability, thus conferring aggressive, basal-like malignant features. In turn, genetic depletion and pharmacological inactivation of SIRT2 reversed stabilization of the SLUG transcription factor and inhibited tumor growth [64]. Moreover, SIRT2 silences a tumor suppressor—arrestin domain-containing 3 (ARRDC3), contributing to the aggressive phenotype of BLBC cells [63,65]. It has been demonstrated that SIRT2 inhibition by TM, a potent SIRT2-specific inhibitor with a broad anticancer effect in numerous human cancer cells as well as mouse models of BC, promotes expression of the NEDD4 E3 Ubiquitin Protein Ligase for c-Myc, causing c-Myc ubiquitination and degradation. Interestingly, TM had a limited influence on normal human cells and tumor-free mice [66]. In turn, RK-9123016, another SIRT2 inhibitor, inhibited the enzymatic activity of SIRT2 with an IC_50_ value of 0.18 μM, but no other human sirtuin members, including SIRT1 and SIRT3, were affected, as well as no activities of zinc-dependent HDACs, including HDAC1 and HDAC6, were affected at 100 μM. Moreover, RK-9123016 reduced the viability of the MCF7 BC cells with IC_50_ = 10 μM, which was lower than IC50 for AGK2 in the MCF7 BC cells (IC_50_ = 66.198 μM) in our studies. Additionally, RK-9123016 exhibited anticancer activity through downregulation of the c-Myc oncoprotein expression and increased the acetylation level of the physiological substrate of SIRT2—eukaryotic translation initiation factor 5A (eIF5A) [67]. Moreover, the SIRT2 protein level was significantly increased in aldehyde dehydrogenase 1-positive (ALDH1+) breast cancer stem cells (CSCs) isolated from primary human breast tumors. NOTCH-induced SIRT2 deacetylation of K353 in ALDH1A1 led to enzymatic activation of SIRT2 and maintained breast CSCs [68]. Bioinformatics analysis demonstrated that three miRNAs, miR-212, miR-375 and miR-655, regulate the bovine SIRT2 gene expression; however, only miR-212 has been shown to have a targeting relationship with SIRT2. MiR-212 targeted and inhibited the expression of the SIRT2 gene to promote lipogenesis in mammary epithelial cell lines. MiR-212 regulated the expression of fatty acid synthetase (FASN) and sterol regulatory element-binding factor 1 (SREBP1) as well as increased the fat content in mammary epithelial cell lines [69].

Inhibition of SIRT2 expression results in the arrest of growth in many types of cancer cells. In this regard, selective SIRT2 inhibitors carry a therapeutic promise in a wide range of tumors, including BC [70]. Here, we determined the responses of different molecular subtypes of BC cells to the AGK2 and PAX treatment. Both these agents administered separately induced inhibition of cell viability in all the tested BC cell lines in a dose-dependent fashion. Interestingly, cytotoxicity of AGK2 was lower in the luminal cells than in the TNBC cells. Surprisingly, the most aggressive HCC1937 *BRCA1* and *p53*-mutated TNBC cells were the most sensitive to the AGK2 treatment. The IC_50_ value for the most resistant MCF7 luminal BC cells was more than 50 times higher than IC_50_ for the HCC1937 TNBC cells. The IC_50_ values for PAX ranged between 1 and 20 nM. Similar to AGK2, a higher IC_50_ value was calculated for the luminal BC cells than for the TNBC cells (except for the mutant HCC1937 BC cells). Interestingly, the HCC1937 BC cells were the most sensitive to the AGK2 treatment and the least sensitive to the PAX treatment. In the study, we also showed the concentration-dependent inhibition of growth after both compounds in a 1:1 ratio combination in all the analyzed BC cell lines. We detected that AGK2 reduced the proliferation of all the luminal and most TNBC cells in a dose-dependent fashion; however, the antiproliferative effect of AGK2 was much weaker than the effect caused by PAX. The combination of PAX with AGK2 reduced BC cell proliferation in all the TNBC (MDA-MB-231, MDA-MB-468, BT-549) cell lines, except for the HCC1937 cells. The T47D and MCF7 cells were the most sensitive for the PAX and AGK2 combined treatment. Similarly to our results, other research groups have demonstrated that AGK2 decreases survival of C6 glioma cells [71] and displays good antiproliferative and cytotoxic activities against glioblastoma (GB) multiforme cancer stem cells (CSCs) [34]. Moreover, AGK2 suppressed the formation of spheres in the CD133-positive cells isolated from GB tissue samples [35]. AGK2 in doses above 30 μM demonstrated growth inhibition of HeLa cervical cancer cells but not of the immortalized HaCaT cells, suggesting that this inhibitor may find application in the treatment of cervical malignancy as well [35].

Based on the cell cycle arrest analysis, we noticed that changes in the cell cycle caused by individual or combined AGK2/PAX treatment depend on the type of the BC cell line. Treatment of the MCF7 luminal BC cells with PAX accumulated the cells in the pre-G1 and G2 phases in a concentration-dependent fashion. Interestingly, incubation of the MCF7 cells with AGK2 caused cell cycle inhibition in the G1 phase. In turn, the concomitant treatment with PAX and AGK2 demonstrated a tendency similar to the PAX treatment—accumulation of cells in pre-G1 and G2 phases. This effect was much more evident in the MCF7 luminal BC cells than in the MDA-MB-468 TNBC cells. In contrast to cell cycle progression, the combined AGK2/PAX treatment slightly increased the percentage of apoptotic cells in all the analyzed BC cell lines, suggesting that AGK2 gently strengthens the effect of PAX. PAX and AGK2 used individually increased the number of cells with active caspase-3 versus control in a dose-dependent fashion. The most significant increase in the number of apoptotic cells after the PAX and AGK2 treatment both separately and in combination was observed in the MCF7 luminal BC cells. Similar results were obtained from other groups, who demonstrated that AGK2 induces growth inhibition in the sub-G0 (pre-G1) phase in glioblastoma cells [35] and in the sub-G0 and G1 phases in cervical cancer cells which were mediated by the decrease in cyclin D1, Cdk4 and Cdk6 expression [36]. AGK2 also induced both necrotic and apoptotic changes in the C6 glioma cells [35]; however, apoptotic cells were not markedly observed after the AGK2 treatment in HeLa cervical cancer cells. LC3B and beclin-1, key autophagy proteins, were also not activated after treatment with AGK2 in this cancer [36]. All these findings suggest that AGK2-caused cell death is cancer-type specific.

To examine the type of pharmacological drug–drug interaction, isobolographic analysis was applied. Isobolography is a restrictive and accurate pharmacodynamic method used to describe the type of interactions between different drugs used in a broad range of doses in in vitro and in vivo settings. Isobolography allows evaluating whether two or more compounds may make a potent combination, improving the effectiveness of treatment [22]. Theoretically, four main types of pharmacological interactions may be distinguished, i.e., supra-additivity/synergy, additivity, sub-additivity/relative antagonism and infra-additivity/absolute antagonism [24].

In the study, we analyzed the efficacy of cotreatment and the types of pharmacological interactions between PAX and AGK2 in the selected BC cell lines to assess the potential application of combinatorial treatment using these two drugs in BC therapy. Based on the isobolographic method, we demonstrated that the combination of PAX and AGK2 at the 1:1 fixed ratio showed a synergistic interaction in the MCF7 luminal BC cells and an additive interaction in the T47D luminal and MDA-MB-231, MDA-MB-468, BT-549 TNBC cells. Interestingly, in the most aggressive HCC1937 BC cells, the combination of AGK2 with PAX revealed an antagonistic interaction, which can be associated with numerous mutations in the *BRCA* and *p53* genes in this cell line. The best type of interaction (synergism) between PAX and AGK2 was shown in the MCF7 luminal BC cells. The MCF7 cells are much less invasive than the TNBC cells, and the treatment of the luminal subtype of BC is much more effective. The concomitant administration of PAX and AGK2 allowed reducing the doses of PAX to achieve a better antiproliferative effect in these BC cells. Combined therapy using these two drugs may be a promising chemotherapy regimen in the therapy of selected subtypes of BC. However, all these findings suggest that the therapy of BC patients with PAX and AGK2 should be highly personalized.

As we mentioned above, isobolography is a very precise method; however, it is not used very often to examine the types of interactions in cancer-related studies. Rather, simple correlations between the investigated compounds are presented, where only a few randomly chosen doses are selected [72]. So far, no studies determining the activity of PAX and AGK2 have been published.

The combination of AGK2 and another sirtuin inhibitor EX-527 exerted an additive antiproliferative effect in glioma cell lines, while in GBM 30P CSC clones these two agents acted synergistically [71]. Interestingly, this combination did not cause cell cycle arrest or induce apoptosis in the U937 human leukemia cell line [34]. AGK2 also demonstrated an additive effect on the cytotoxicity of lapatinib in the sensitive 6–10B and the resistant 5–8F nasopharyngeal carcinoma (NPC) cell lines [37]. It has also been demonstrated that dichloroacetic acid (DCA) synergizes with AGK2 in non-small cell lung cancer (NSCLC). The cotreatment of DCA with AGK2 effectively reduced survival of both H1299 and A549 cell lines by 80–90%. AGK2 increased the acetylation of lysines and decreased the phosphorylation of serines in pyruvate dehydrogenase alpha 1 (PDHA1), which enabled AGK2 to synergize with DCA. AGK2 induced metabolic remodeling from glycolysis to the mitochondrial oxidative phosphorylation system (OXPHOS), including decreased lactate production and glucose consumption as well as an increased oxygen consumption rate (OCR) and reactive oxygen species (ROS) generation. AGK2 in combination with DCA meaningfully promoted cancer inhibition in comparison with monotherapy, providing a rationale for the use of these drugs in combination in order to inhibit proliferation of NSCLC cells [38].

Concurrent administration of AGK2 and standard cytostatics can be a new modern strategy to enhance the effectiveness of the currently used cancer patients’ therapeutic methods and ensure successful elimination of BC cells. Synergistic or additive pharmacological interactions of the tested active agents in most of the analyzed BC cells as well as the promising 2D in vitro results strongly suggest an application of these drugs in combination in more advanced preclinical models, e.g., in 3D organoid models or animal xenografts.

## 5. Conclusions

**Figure 20:**
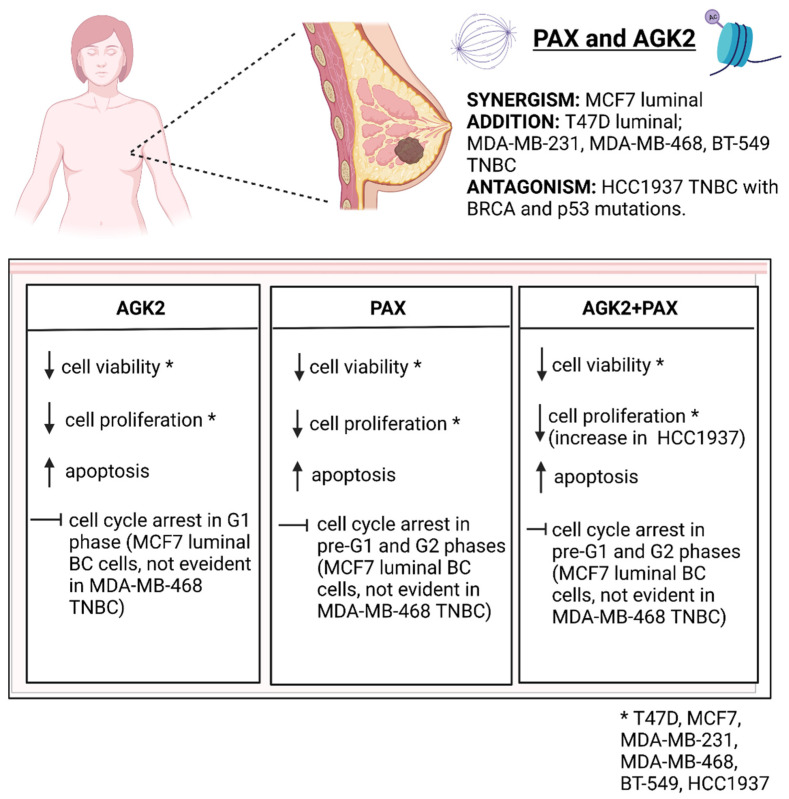
Summary of the PAX/AGK2 activity in the T47D, MCF7, MDA-MB-231, MDA-MB-468, BT-549, HCC1937 breast cancer (BC) cells (decrease; increase; TNBC—triple-negative breast cancer). Created with www.biorender.com (accessed on 31 March 2022).

To sum up, our studies demonstrate that AGK2 applied with PAX could be used in the therapy of some subtypes of BC (Figure 20) in order to reduce their doses in relation to those administered individually and consequently improve their antitumor activities. 

The use of AGK2 and PAX in combination could principally eliminate resistance to PAX in BC patients and reduce the doses of PAX to limit the adverse effects of this drug. Given the fact that PAX can induce heavy side effects, the use of lower doses of PAX together with AGK2 appears a promising therapeutic approach in personalized BC therapy. Concurrent administration of the tested drugs may be a new interesting strategy in order to increase the effectiveness of the currently used anticancer regimens and more effectively kill BC cells. However, the molecular mechanisms of AGK2 with PAX at the cellular level require further research. 

## Figures and Tables

**Table 1:** The detailed characteristics of BC cell lines used in the study.

	T47D	MCF7	MDA-MB-231	MDA-MB-468	BT-549	HCC1937
**Tissue**	Breast, mammary gland	Breast, mammary gland	Breast, mammary gland	Breast, mammary gland	Breast, mammary gland	Breast, duct, mammary gland
**Disease**	Carcinoma, ductal	Adenocarcinoma	Adenocarcinoma	Adenocarcinoma	Carcinoma, ductal	Carcinoma, ductal *(BRCA1* and *p53*-mutated)
**Age (years)**	54	69	51	51	72	23
**Gender**	Female	Female	Female	Female	Female	Female
**Metastases**	Pleural effusion	Pleural effusion	Pleural effusion	Pleural effusion	Three of the seven regional lymph nodes	Pleural effusion
**Cell type**	Epithelial	Epithelial	Epithelial	Epithelial	Epithelial	Epithelial
**Growth properties**	Adherent	Adherent	Adherent	Adherent	Adherent	Adherent
**Receptor expression**	**Estrogen receptor,** **progesterone receptor**	**Estrogen receptor,** **progesterone receptor**	**TNBC**	**TNBC**	**TNBC**	**TNBC**
**References**	[45]	[46]	[47]	[48]	[49]	[50]

**Table 2:** IC_50_ ± SEM for AGK2 and paclitaxel (PAX) for the T47D, MCF7, MDA-MB-231, MDA-MB-468, BT-549 and HCC1937 BC cells. N—number of items; SR—slope function ratio (S_PAX_/S_AGK2_); f ratio SR—factor for the slope function ratio; NP—not parallel; P—parallel. The test for parallelism of the two concentration–effect curves for PAX and AGK2 was performed according to Litchfield and Wilcoxon [58].

Cell Line	Drug	IC_50_ (μM)	N	SR	f Ratio SR	Parallelism
T47D	PAX	0.006095 ± 0.002072	72	23.338	7.910	NP
T47D	AGK2	17.727 ± 11.868	120			
MCF7	PAX	0.015669 ± 0.006457	72	3.607	3.600	NP
MCF7	AGK2	66.198 ± 32.084	120			
MDA-MB-231	PAX	0.001712 ± 0.000543	72	1.449	2.462	P
MDA-MB-231	AGK2	6.111 ± 2.007	96			
MDA-MB-468	PAX	0.004546 ± 0.001148	72	1.389	1.659	P
MDA-MB-468	AGK2	4.562 ± 1.214	96			
BT-549	PAX	0.002783 ± 0.000547	48	7.702	2.021	NP
BT-549	AGK2	16.108 ± 6.243	120			
HCC1937	PAX	0.018604 ± 0.008392	72	1.926	5.318	P
HCC1937	AGK2	1.326 ± 0.644	96			

**Table 3:** Isobolographic analysis for nonparallel concentration–effect curves lines between PAX and AGK2 in the T47D, MCF7 and BT-549 BC cells. The results are presented as the IC_50_ values in μM ± SEM for the two-drug mixtures determined experimentally (IC_50 mix_) and theoretically computed (IC_50 add_) from the equations of additivity, which blocked proliferation in 50% of the tested cells; n _mix_—total number of items for the experimental mixture; n _add_—total number of items calculated for the additive mixture of two examined drugs (n _add_ = n__PAX_ + n__AGK2_ – 4); L-IC_50 add_—lower additive value; U-IC_50 add_—upper additive value. Note: * *p* < 0.05 vs. the respective IC_50 add_ value (Student’s *t*-test with Welch’s correction).

Cell Line	IC_50 mix_ (μM)	n _mix_	L-IC_50 add_ (μM)	n _add_	U-IC_50 add_ (μM)	n _add_	Interaction
T47D	7.491 ± 1.729	120	4.668 ± 4.162	188	13.112 ± 4.820	188	Additivity
MCF7	3.618 ± 0.873 *	120	25.453 ± 10.865	188	40.841 ± 10.895	188	Synergy
BT-549	6.805 ± 1.570	120	4.731 ± 2.190	164	11.378 ± 2.862	164	Additivity

**Table 4:** Isobolographic analysis for parallel CECs between PAX and AGK2 at the 1:1 ratio in the MDA-MB-231, MDA-MB-468 and HCC1937 cells. The results are IC_50_ values (in μM ± SEM) for the two-drug mixtures determined experimentally (IC_50 mix_) and theoretically computed (IC_50 add_) from the equation of additivity that blocked proliferation in 50% of the tested cells; n _mix_—total number of items for the experimental mixture; n _add_—total number of items calculated for the additive mixture of the examined drugs (n _add_ = n__PAX_ + n__AGK2_ – 4); * *p* < 0.05 vs. the respective IC_50 add_ value.

Cell Line	IC_50 mix_ (μM)	n _mix_	IC_50 add_ (μM)	n _add_	Interaction
MDA-MB-231	2.661 ± 0.450	120	3.056 ± 1.003	164	Additivity
MDA-MB-468	2.129 ± 0.299	120	2.283 ± 0.608	164	Additivity
HCC1937	3.827 ± 1.476 *	120	0.673 ± 0.326	164	Antagonism

## Data Availability

Publicly available datasets were analyzed in this study. These data can be found here: http://www.cbioportal.org/public-portal, https://www.proteinatlas.org/public-portal, https://cancer.sanger.ac.uk/cosmic, https://biit.cs.ut.ee/methsurv/; accessed on 15 February 2022.
